# Correlation of systemic immune-inflammation Index with surgical necrotizing enterocolitis

**DOI:** 10.3389/fped.2022.1044449

**Published:** 2022-11-07

**Authors:** Wei Feng, Jinping Hou, Chenzhu Xiang, Xiaohong Die, Jing Sun, Zhenhua Guo, Wei Liu, Yi Wang

**Affiliations:** Department of General & Neonatal Surgery, Children's Hospital of Chongqing Medical University, National Clinical Research Center for Child Health and Disorders, Ministry of Education Key Laboratory of Child Development and Disorders, Chongqing Key Laboratory of Pediatrics, Chongqing, China

**Keywords:** necrotizing enterocolitis, systemic immune-inflammation index, surgery, mortality, neonate

## Abstract

**Background:**

Systemic Immune-Inflammation Index (SII), known as an easy, economical and useful marker, correlates with the severity of inflammatory response. However, the usefulness of SII in necrotizing enterocolitis (NEC) remains unclear. Therefore, we evaluated the correlation of SII at NEC diagnosis and subsequent surgery.

**Methods:**

Retrospective review of 131 neonates with NEC in a tertiary-level pediatric referral hospital was conducted with assessments of demographic data, general blood examination results at NEC diagnosis, treatment strategies and clinical outcomes. The receiver operating characteristic (ROC) curve determined the optimal cut-off values of SII, platelet-to-lymphocyte ratio (PLR), and neutrophil-to-lymphocyte ratio. Univariate/multivariate logistic regression analysis and ROC curve were conducted to evaluate the predictive significance of SII in identifying the patients who eventually received surgery. Additionally, NEC-related deaths were assessed.

**Results:**

Overall, 49 (37.4%) cases received surgical intervention and mortality was 12.3% (14/131). The area under ROC curve of SII at NEC diagnosis to predict subsequent surgery was 0.833 (optimal cut-off value: 235.85). The SII value in surgical intervention group was significantly higher than that in medical treatment group (332.92 ± 158.52 vs. 158.84 ± 106.82, *P* < 0.001). Independent influencing factors for surgical NEC were SII (95% confidence interval [CI]: 4.568∼36.449, odds ratio [OR]:12.904, *P* < 0.001) and PLR (95% CI: 1.071∼7.356, OR:2.807, *P* = 0.036). SII ≤ 235.85 could identify patients at high risk for surgery, with 87.76% sensitivity, 73.17% specificity, outperformed PLR. Furthermore, mortality was significantly higher in patients with SII ≤ 235.85 than those with SII > 235.85 (20.0% vs. 1.5%, *P* < 0.001).

**Conclusion:**

SII and PLR at NEC diagnosis were independent influencing factors for subsequent surgery. SII ≤ 235.85 may be a useful predictive marker for the identification of surgical NEC and mortality.

## Introduction

Necrotizing enterocolitis (NEC) is one of the most common and critical neonatal gastrointestinal emergencies with significant morbidity and mortality (1). Over the past several decades, selection of treatment strategies for NEC remains a challenge for pediatric surgeons and neonatologists. When inflammation and intestinal injury are limited, NEC can be treated medically, includes bowel rest and careful use of antibiotics, but surgery is needed if the patient continue to deteriorate after medical treatment or develop bowel perforation (2). Epidemiologic data revealed that about 30% of neonates with NEC may require surgical intervention with an overall case-fatality rate of 15% (3). It is noteworthy that surgical NEC poses potentially devastating condition, e.g., higher mortality than conservative treatment, short bowel syndrome, failure to thrive, and neurodevelopmental impairment (4). Being able to identify surgical NEC early allows the surgeon or neonatologist to optimize referral and treatment strategies, and potentially lead to improve clinical outcomes (5). Furthermore, this is also conducive to fully communicate with family members about the disease, as well as target limited medical resources for those at the highest risk. Nevertheless, few studies have specifically looked at early predictors of surgical NEC. Consequently, simple and effective methods to estimate surgical NEC are currently of interest.

Inflammatory assessment indexes based on blood routine results, as white blood cell count (WBC), neutrophil count (NE), neutrophil-lymphocyte ratio (NLR), platelet-lymphocyte ratio (PLR), systemic immune-inflammation index (SII), are widely used to assess the severity or clinical outcomes of inflammation related diseases (6–8). As we all know, platelets, neutrophils, and lymphocytes are all involved in the inflammatory response as inflammatory cells, and the composite index SII developed based on them can comprehensively assess the balance between inflammatory and immunological responses (9). The inflammation is closely related to the occurrence and development of tumor (10). Studies have shown that for malignant tumors, included hepatocellular carcinoma (HCC), gastric carcinoma, patients with high preoperative SII value (grouped by the optimal cut-off point) indicates a worse clinical outcome (9, 11). SII can be used as an effective index to assess the degree of inflammation. For example, SII could effectively identify the severity of acute pancreatitis, outperformed NLR and PLR (12). Compared with mild COVID-19 patients, the severe had significantly higher SII value (13). Usually, the increase of SII value often suggests the aggravation of inflammation and poor clinical outcome, with considerable sensitivity and specificity.

The calculation of SII is based on complete blood count technology, with the advantages of low cost, simple measurement, quick results and high repeatability. However, there is a lack of studies on the application of SII in children, especially neonates. NEC is a severe inflammatory intestinal disease associated with the imbalance in the maturation of intestinal innate and adaptive immune defense mechanisms (14, 15). Based on this, we hypothesize that SII might be associated with surgical risk in patients with NEC. The present study investigated the predictive significance of SII for the identification of surgical NEC and NEC-related death.

## Materials and methods

### Study design and setting

This was a a single institution, retrospective study conducted at the Gastrointestinal Neonatal Surgery Department of Children's Hospital affiliated Chongqing Medical University, a tertiary pediatric hospital and National Children's Medical Center in China, between December 2019 and October 2020. It was performed after the study protocol was approved by the Institutional Research Ethics Board of Children's Hospital affiliated Chongqing Medical University (Date: 09.28.2021/No: 329). This is a teaching and research hospital where medical students and residents collect the clinical data of patients using standardized forms under the supervision of senior pediatricians and professors.

### Study population

The clinical data of patients with NEC (Bell's stage ≥ II) admitted to our department during the study period were analyzed retrospectively. The inclusion criteria were: (1) patients were diagnosed with NEC according to the radiological evidence (i.e., pneumatosis intestinalis) and presence of one or more clinical fndings (i.e., abdominal distension, bilious/bloody aspirates, blood per rectum, abdominal tenderness, abdominal wall erythema/discoloration or abdominal mass) (16), and (2) those who had complete medical records and postoperative follow-up data. The exclusion criteria were as follows: (1) patients with digestive system deformity that increase the risk of secondary NEC, like Hirschsprung's disease, (2) those received surgery for intestinal perforation due to meconium-related ileus, (3) those with hematologic diseases, (4) those identified as “spontaneous isolated intestinal perforation” by the operating surgeon and histologically, (5) those with incomplete clinical data.

### Surgical intervention

Surgical intervention was defined as performance of exploratory laparotomy or placement of a peritoneal drain. Indications for surgery included evidence of intestinal perforation (free intraperitoneal air on radiological examination) and/or clinical deterioration (increasing abdominal distension, erythema, discoloration and tenderness) despite maximum conservative treatment (16). It is worth noting that the relative surgical indications of NEC are still controversial, such choices were dictated by the clinical judgment of the pediatric surgeon in coordination with neonatologist. According to our clinical practice, some patients did not receive surgical intervention during the acute process of NEC, but needed surgery due to later complications, which actually had a causal relationship (17, 18). So, to improve the clinical practicability, this study also included the patients who received medical treatment for NEC but had surgery for later complications of NEC including persistent intestinal obstruction or stricture during 3-month follow-up period (16).

### Data collection

In order to improve the convenience of clinical use, the variables for inclusion were carefully selected to make sure the parsimony of the final models. The data collected for patients included (1) general demographic data: gender, gestational age, birth weight, small for gestational age, pregnancy, Apgar score at 5 min, postmenstrual age at NEC diagnosis, red blood transfusion within 48 h before NEC diagnosis, maternal age and vaginal delivery; (2) general blood examination results measured at the time of or at the earliest time after the diagnosis of NEC (within 12 h): WBC, NE, lymphocyte count (LY), monocyte count (MO), hemoglobin (HB), platelet count (PLT), and C-reactive protein (CRP); (3) treatment strategies: surgical intervention or conservative treatment; (4) clinical outcomes: survival or mortality during hospitalization or within the 3-month follow-up period after discharge. It is common in applied epidemiological and clinical research to convert numerical variables into categorical variables by grouping values into categories (19, 20). So, referring to relevant literatures and considering the clinical practice, we recorded the general demographic data as categorical variables (21–25).

### Definitions

The results of general blood examination at NEC diagnosis were collected, and SII, PLR, and NLR were calculated as: SII = PLT (10^9^/L) × NE (10^9^/L) / LY (10^9^/L), PLR = PLT (10^9^/L) / LY (10^9^/L), and NLR = NE (10^9^/L) / LY (10^9^/L) (26). To make the analysis simple and easy to application, the patients were divided into groups according to the optimal cut-off values of SII, PLR and NLR, and the correlations of these indexes with general demographics were analyzed.

### Statistical analysis

SPSS 22.0 and GraphPad Prism 8.0 were used for statistical analysis. Categorical data were expressed by n (%), and compared using the Chi-square or Fisher's exact test. Numerical data were tested for normality using Shapiro-Wilk test. Based on whether or not it conforms to normal distribution, numerical variables were expressed as the mean ± standard deviation (SD) or median and interquartile range (IQR) and were compared using Student's t-test or Mann–Whitney U test, as appropriate. We dichotomized numerical variables of SII, PLR, and NLR on threshold calculated based on receiver operating characteristic (ROC) curve and Youden index (Youden index = sensitivity + specificity −1) yielding the best performance of prediction. Variables on univariate analyses with statistical significance (included LY, PLT, CRP, Low-SII, Low-PLR, and Low-NLR) were included in multivariate logistic regression model (forward conditional method) in order to determine risk factors predictive for surgical NEC using odds ratios (OR) with 95% confidence intervals (CI). Moreover, ROC curve analysis was performed to evaluate the predictive abilities of interested factors for surgical NEC and mortality during 3-month follow-up period. Usually, factor with an area under the ROC curve (AUC) above 0.70 was considered to be useful, while an AUC between 0.80 and 0.90 indicated great diagnostic accuracy (27). *P* < 0.05 was considered statistically significant.

## Results

### General data

**Figure 1:**
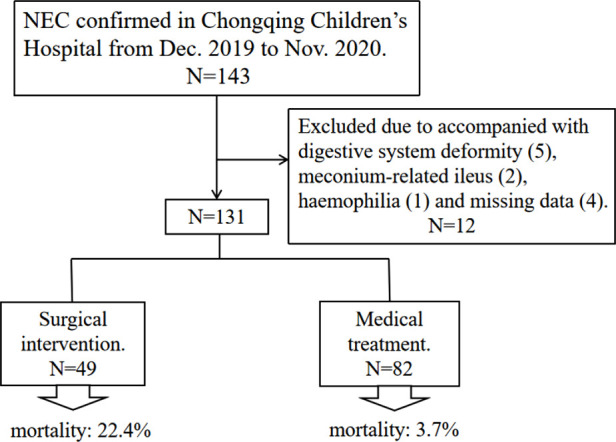
Flow chart of the study population.

Figure 1 shows a flow diagram for the inclusion and exclusion of patients in this study. The entire number of patients met the inclusion and exclusion criteria during the time frame was 131, including 64 females (48.9%) and 67 males (51.1%), with the mean age at NEC diagnosis of 13.6 days. Among them, 49 cases (37.1%) received surgical intervention within follow-up period, including 41 cases received surgery for primary NEC and 8 cases for later complications, and medical treatment was performed in 82 cases (62.9%). A total of 11 deaths were reported in 49 surgical patients (22.4%), and the overall mortality was 12.3%. 38 patients received staged surgery (early exploratory laparotomy + enterostomy and followed by closure of the enterostomy), and 1 patient underwent unplanned reoperation.

### Determination for SII, PLR, and NLR

**Figure 2:**
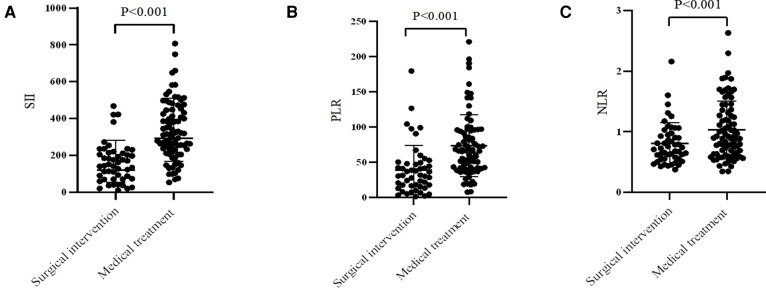
Comparison of (**A**) SII, (**B**) PLR and (**C**) NLR values in different treatment strategies. SII, systemic immune-inflammation index; PLR, platelet-to-lymphocyte ratio; NLR, neutrophil-to-lymphocyte ratio.

**Figure 3:**
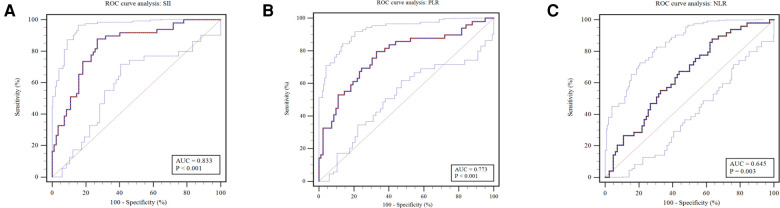
Receiver operating characteristic curves were plotted to determine the optimal cut-off value for (**A**) SII, (**B**) PLR and (**C**) NLR. SII, systemic, immune-inflammation index; PLR, platelet-to-lymphocyte ratio; NLR, neutrophil-to-lymphocyte ratio.

For the comparative results of SII, PLR, and NLR (Figure 2), patients receiving surgical intervention were significantly associated with an decreased SII (mean: 158.84 vs. 332.92, *P* < 0.001), PLR (median: 31.12 vs. 65.82, *P* < 0.001), and NLR (median: 0.73 vs. 0.91, *P* < 0.001). The above numerical variables were dichotomized using ROC curve, which is displayed in Figure 3. The optimal cut-off value was determined based on the maximum combination of sensitivity and specificity (maximum Youden index). This led to the following cut-off values: SII: 235.85, PLR: 50.17, NLR: 1.09. Based on these cut-off values, the patients were divided into Low-SII group (SII ≤ 235.85, *n* = 65) and High-SII group (SII > 235.85, *n* = 66), Low-PLR group (PLR ≤ 50.17, *n* = 65) and High-PLR group (PLR > 50.17, *n* = 66), and Low-NLR group (NLR ≤ 1.09, *n* = 95) and High-NLR group (NLR > 1.09, *n* = 36).

### Correlations of SII, PLR, and NLR with general demographics of neonates with NEC

The level of SII was not correlated with the general demographics of NEC patients, including gender, gestational age, birth weight, small for gestational age, pregnancy, apgar at 5 min, age at NEC diagnosis, blood transfusion, maternal age, or vaginal delivery (all *P* > 0.05), and neither did NLR. PLR was associated with pregnancy and Apgar at 5 min (both *P* < 0.05), but not with gender, gestational age, birth weight, small for gestational age, age at NEC diagnosis, blood transfusion, maternal age, or vaginal delivery (all *P* > 0.05). The analysis results were shown in Table 1.

**Table 1:** Correlations of SII, PLR, and NLR with general demographics of neonates with NEC.

Variables	High-SII	Low-SII	*P* value	High-PLR	Low-PLR	*P* value	High-NLR	Low-NLR	*P* value
Case (*n*)	66	65		66	65		36	95	
Gender (*n*/%)^a^			0.602			0.728			1.000
Male	34 (51.5)	30 (46.2)		31 (47.0)	33 (50.8)		18 (50.0)	46 (48.4)	
Female	32 (48.5)	35 (53.8)		35 (53.0)	32 (49.2)		18 (50.0)	49 (51.6)	
Gestational age (weeks, *n*/%)^a^			0.277			0.179			0.529
<28	2 (3.0)	6 (9.2)		3 (4.5)	5 (7.7)		2 (5.6)	6 (6.3)	
28∼<32	16 (24.2)	18 (27.7)		16 (24.2)	18 (27.7)		12 (33.3)	22 (23.2)	
32∼<34	18 (27.3)	12 (18.5)		21 (31.8)	9 (13.8)		10 (27.8)	20 (21.1)	
34∼<37	19 (28.8)	23 (35.4)		18 (27.3)	24 (36.9)		8 (22.2)	34 (35.8)	
≥37	11 (16.7)	6 (9.2)		8 (12.1)	9 (13.8)		4 (11.1)	13 (13.7)	
Birth weight (grams, *n*/%)^a^			0.058			0.694			0.868
<1000	5 (7.6)	9 (13.8)		6 (9.1)	8 (12.3)		4 (11.1)	10 (10.5)	
1000∼<1500	15 (22.7)	15 (23.1)		15 (22.7)	15 (23.1)		10 (27.8)	20 (21.1)	
1500∼<2500	30 (45.5)	36 (55.4)		32 (48.5)	34 (52.3)		17 (47.2)	49 (51.6)	
≥2500	16 (24.2)	5 (7.7)		13 (19.7)	8 (12.3)		5 (13.9)	16 (16.8)	
Small for gestational age (*n*/%)^a^			0.657			0.375			0.463
Yes	11 (16.7)	13 (20.0)		10 (15.2)	14 (21.5)		5 (13.9)	19 (20.0)	
No	55 (83.3)	52 (80.0)		56 (84.8)	51 (78.5)		31 (86.1)	76 (80.0)	
Pregnancy (*n*/%)^a^			1.000			0.039			0.165
Single	55 (83.3)	46 (70.8)		56 (84.8)	45 (69.2)		31 (86.1)	70 (73.7)	
Multiple	11 (16.7)	19 (29.2)		10 (15.2)	20 (30.8)		5 (13.9)	25 (26.3)	
Apgar at 5 min (*n*/%)^a^			0.195			0.030			0.473
<7	17 (25.8)	10 (15.4)		19 (28.8)	8 (12.3)		9 (25.0)	18 (18.9)	
≥7	49 (74.2)	55 (84.6)		47 (71.2)	57 (87.7)		27 (75.0)	77 (81.1)	
Age at NEC diagnosis (days, *n*/%)^a^			0.074			0.520			1.000
≤7	3 (4.55)	11 (16.9)		5 (7.6)	9 (13.8)		4 (11.1)	10 (10.5)	
>7∼14	35 (53.0)	31 (47.7)		35 (53.0)	31 (47.7)		18 (50.0)	48 (50.5)	
>14	28 (42.45)	23 (35.4)		26 (15.4)	25 (38.5)		14 (38.9)	37 (38.9)	
Blood transfusion (*n*/%)^a^			0.575			0.350			0.141
Yes	19 (28.8)	22 (33.8)		18 (27.3)	23 (35.4)		15 (41.7)	26 (27.4)	
No	47 (71.2)	43 (66.2)		48 (72.7)	42 (64.6)		21 (58.3)	69 (72.6)	
Maternal age (*n*/%)^a^			0.809			1.000			0.423
<35	55 (83.3)	56 (86.2)		56 (84.8)	55 (84.6)		29 (80.6)	82 (86.3)	
≥35	11 (16.7)	9 (13.8)		10 (15.2)	10 (15.4)		7 (19.4)	13 (13.7)	
Vaginal delivery (*n*/%)^a^			0.475			1.000			0.841
Yes	28 (42.4)	23 (35.4)		26 (39.4)	25 (38.5)		15 (41.7)	36 (37.9)	
No	38 (57.6)	42 (64.6)		40 (60.6)	40 (61.5)		21 (58.3)	59 (62.1)	

^a^ Chi-square test.

### Univariate/multivariate regression analyses of patients receiving surgical intervention

The general demographics, general blood examination results of different treatment strategies for NEC are listed in Tables 2,3. No significant differences in gender, gestational age, birth weight, small for gestational age, pregnancy, Apgar score at 5 min, age at NEC diagnosis, blood transfusion, maternal age, vaginal delivery, WBC, NE, MO, and HB between surgical intervention and medical treatment groups (all *P* > 0.05). Univariate analysis showed LY, PLT, and CRP were the potential influencing for surgical NEC. Furthermore, the surgical intervention rate was remarkably higher in the Low-SII group than in the High-SII group (87.8% vs. 26.8%, *P* < 0.001), in the Low-PLR group than in the High-PLR group (77.6% vs. 32.9%, *P* < 0.001), and in the Low-PNI group than in High-NLR group (87.8% vs. 63.4%, *P* = 0.003). Multivariate regression analysis is conducive to balancing the interactions between variables and comparable so that non-random grouping data can be used to study the relationship between trial factors and dependent variable, and obtain more reliable research results. So, factors with statistical significance (*P* < 0.05) in the univariate analysis were included in multivariate regression analysis (16). It was found that Low-SII (OR = 12.904, 95% CI: 4.568∼36.449, *P *< 0.001), and Low-PLR (OR = 2.807, 95% CI: 1.071∼7.356, *P* = 0.036) were independent risk factors for surgical NEC, whereas NLR was not a independent risk factors (Table 4). In other words, compared with High-SII, patients with Low-SII had a 12.904 times higher chance of receiving surgical intervention. Diagnosis of collinearity for the above two variables was performed, and the variance inflation factor (VIF) and tolerance (TOL) were 1.354 (VIF ≤ 10) and 0.738 (TOL ≥ 0.1), respectively, suggesting that there was no multiple collinearity relationship.

**Table 2:** Univariate analysis of general demographics on different treatment strategies for NEC.

Variables	Overall (*N* = 131)	Surgical intervention (*N* = 49)	Medical treatment (*N* = 82)	*P* value
Gender (*n*/%)^a^				0.588
Male	67 (51.1)	22 (44.9)	42 (51.2)	
Female	64 (48.9)	27 (55.1)	40 (48.8)	
Gestational age (weeks, *n*/%)^a^				0.167
<28	8 (6.1)	5 (10.2)	3 (3.7)	
28∼<32	34 (26.0)	14 (28.6)	20 (24.4)	
32∼<34	30 (22.9)	6 (12.2)	24 (29.3)	
34∼<37	42 (32.1)	17 (34.7)	25 (30.5)	
≥37	17 (13.0)	7 (14.3)	10 (12.2)	
Birth weight (grams, *n*/%)^a^				0.348
<1000	13 (9.9)	8 (16.3)	6 (7.3)	
1000∼<1500	31 (23.7)	12 (24.5)	18 (22.0)	
1500∼<2500	66 (50.4)	21 (42.9)	45 (54.9)	
≥2500	21 (16.0)	8 (16.3)	13 (15.9)	
Small for gestational age (*n*/%)^a^				0.360
Yes	24 (18.3)	11 (22.4)	13 (15.9)	
No	107 (81.7)	38 (77.6)	69 (84.1)	
Pregnancy (*n*/%)^a^				0.133
Single	101 (77.1)	34 (69.4)	67 (81.7)	
Multiple	30 (22.9)	15 (30.6)	15 (18.3)	
Apgar score at 5 min (n/%)^a^				0.188
<7	27 (20.6)	7 (14.3)	20 (24.4)	
≥7	104 (79.4)	42 (85.7)	62 (75.6)	
Postmenstrual age at NEC diagnosis (days, *n*/%)^a^				0.251
≤7	14 (10.7)	8 (16.3)	6 (7.3)	
>7∼14	66 (50.4)	22 (44.9)	44 (53.7)	
>14	51 (38.9)	19 (38.8)	32 (39.0)	
Blood transfusion (*n*/%)^a^				0.176
Yes	41 (31.3)	19 (38.8)	22 (26.8)	
No	90 (67.7)	30 (61.2)	60 (73.2)	
Maternal age (*n*/%)^a^				0.617
<35	111 (84.7)	43 (87.8)	68 (82.9)	
≥35	20 (15.3)	6 (12.2)	14 (17.1)	
Vaginal delivery (*n*/%)^a^				0.465
Yes	51 (38.9)	17 (34.7)	34 (41.5)	
No	80 (61.1)	32 (65.3)	48 (58.5)	

^a^ Chi-square test.

**Table 3:** Laboratory data comparison between surgical intervention and medical treatment.

Blood test parameters	Overall (*N* = 131)	Surgical intervention (*N* = 49)	Medical treatment (*N* = 82)	*P* value
General blood examination				
WBC (×10^9^/L)^a^	11.86 (9.04, 16.91)	12.48 (9.48, 17.94)	11.64 (8.95, 14.79)	0.164
NE (×10^9^/L)^c^	5.47 ± 3.00	5.44 ± 2.79	5.49 ± 3.13	0.921
LY (×10^9^/L)^c^	6.48 ± 3.69	7.48 ± 4.25	5.89 ± 3.18	0.016
MO (×10^9^/L)^a^	0.87 (0.56, 1.33)	0.88 (0.57, 1.34)	0.87 (0.56, 1.30)	0.853
HB (g/L)^a^	138.0 (116.0,163.0)	136.0 (111.5,155.5)	140.0 (119.0,164.0)	0.265
PLT (×10^9^/L)^a^	292.0 (165.0,408.0)	171.0 (91.5,310.5)	328.0 (255.0,431.3)	<0.001
CRP (mg/L)^b^				0.022
<8	81 (59.6)	24 (49.0)	57 (69.5)	
8∼20	20 (15.8)	8 (16.3)	12 (14.6)	
>20∼50	21 (16.7)	10 (20.4)	9 (13.4)	
>50	9 (7.9)	7 (14.3)	2 (2.4)	
Combination marker				
Low-SII^b^				<0.001
Yes	65 (49.6)	43 (87.8)	22 (26.8)	
No	66 (50.4)	6 (12.2)	60 (73.2)	
Low-PLR^b^				<0.001
Yes	65 (49.6)	38 (77.6)	27 (32.9)	
No	66 (50.4)	11 (22.4)	55 (67.1)	
Low-NLR^b^				0.003
Yes	95 (72.5)	43 (87.8)	52 (63.4)	
No	36 (27.5)	6 (12.2)	30 (36.6)	

^a^ Values are presented as medians (IQR) and used Mann-Whitney U test.

^b^ Chi-square test.

^c^ Values are presented as mean ± standard deviation and used Student's t test.

WBC, white blood cell count; NE, neutrophil count; LY, lymphocyte count; MO, monocyte count; HB, hemoglobin; PLT, platelet count; CRP, C-reactive protein.

**Table 4:** Multivariate logistic regression analysis for surgical NEC.

Variables	*β*	SE	Wald	OR	95% CI	*P* value
Low-SII	2.558	0.530	23.303	12.904	4.568∼36.449	<0.001
Low-PLR	1.032	0.492	4.408	2.807	1.071∼7.356	0.036
Constant	−2.640	0.479	30.362	0.071	-	-

β, regression coefficient; SE, standard error; OR, odds ratio; 95% CI, 95% confidence interval; NEC, necrotizing enterocolitis; SII, systematic immuno-inflammatory index; PLR, platelet-to-lymphocyte ratio.

### Correlations of low-SII and low-PLR with surgical NEC

**Figure 4:**
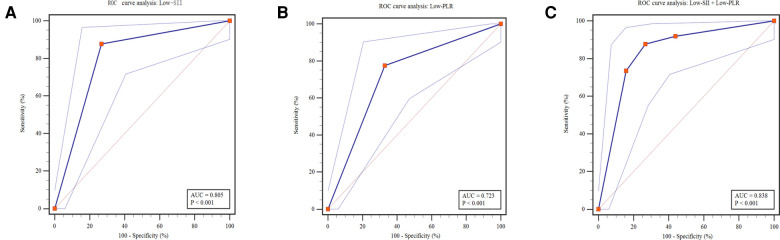
Predictive assessment of Low-SII, Low-PLR and both for patients receiving surgery with ROC curve analysis. ROC, receiver operating characteristics; AUC, area under the curve; SII, systemic immune-inflammation index; PLR, platelet-to-lymphocyte ratio.

Of the 131 patients, 49 (37.4%) cases received surgical intervention. The predictive values of Low-SII and Low-PLR are shown in Figure 4 and Table 5. ROC curve analysis of Low-SII and Low-PLR resulted in AUCs of 0.805 (95% CI: 0.726–0.883, *P* < 0.001) and 0.723 (95% CI: 0.633–0.814, *P* < 0.001), respectively. The Low-SII showed a clearly better predictive performance for identifying the patients who received surgical intervention than Low-PLR, with 87.76% sensitivity, 73.17% specificity, 66.15% positive predictive value (PPV) and 90.91% negative predictive value (NPV). It suggested that patients with Low-SII at NEC diagnosis were considered to be more likely to received surgery. To explore whether the combination of Low-SII and Low-PLR had unexpected prediction efficiency for surgical NEC, we referred the Enter method (*P* = Expi∑BiXi/1 + Exp∑BiXi) to establish prediction model based on the two factors (28). ROC curve (Figure 4) analysis of prediction model resulted in an AUC of 0.838 (95% CI: 0.764–0.897, *P* < 0.001). The predictive values of prediction model in the sample were 87.76% sensitivity, 73.17% specificity, as did the Low-SII alone.

**Table 5:** Predictive value of Low-SII and Low-PLR for surgery.

Variables	Sensitivity (%)	Specifificity (%)	PPV (%)	NPV (%)	+LR	−LR
Low-SII	87.76	73.17	66.15	90.91	3.27	0.17
Low-PLR	77.55	67.07	58.46	83.33	2.36	0.33
Low-SII + Low-PLR	87.76	73.17	66.15	90.91	3.27	0.17

SII, systematic immuno-inflammatory index; PLR, platelet-to-lymphocyte ratio; PPV, positive predictive value; NPV, negative predictive value; +LR, positive likelihood ratio; -LR, negative likelihood ratio.

### Low-SII, low-PLR and surgical intervention provided a reference for clinical outcome of NEC

**Figure 5:**
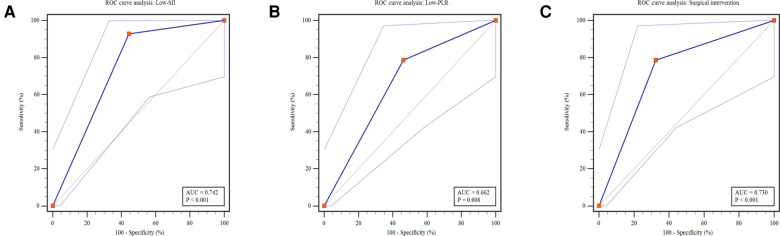
Predictive analysis of outcome for Low-SII, Low-PLR and surgical intervention. ROC, receiver operating characteristics; AUC, area under the Curve; SII, systemic immune-inflammation index; PLR, platelet-to-lymphocyte ratio.

**Figure 6:**
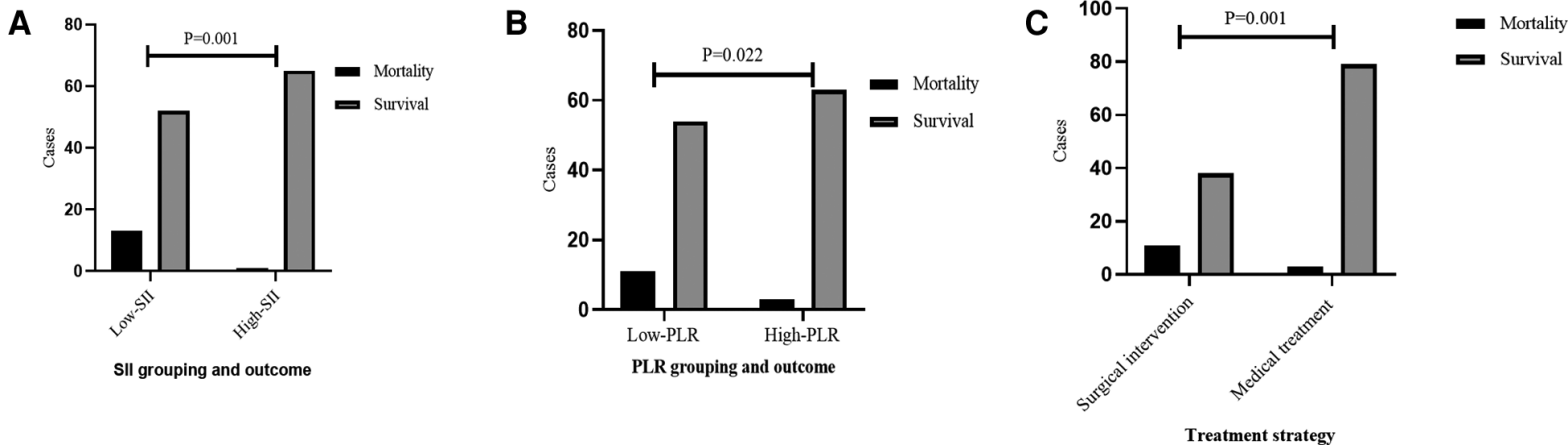
The number of patients who died and survived in different subgroups.

To provide reference for optimizing the allocation of medical resources and guide parental counselling, Low-SII, Low-PLR and surgical intervention was applied to predict the clinical outcomes of NEC. During hospitalization or within the 3-month follow-up period after discharge, 14 deaths were reported in 131 patients (22.4%). The mortality was remarkably higher in the Low-SII group than in the High-SII group (20.0% vs. 1.5%, *P* = 0.001, Figure 5A), and in the Low-PLR group than in the High-PLR group (16.9% vs. 4.5%, *P* < 0.022, Figure 5B). Furthermore, it was distinctly higher in surgical intervention group than in medical treatment group (22.4% vs. 3.7%, *P* < 0.001, Figure 5C), which indicates that patients with NEC who receive surgery were more likely to have poor clinical outcome, consistent with the findings of other reports. It was found that the AUCs of Low-SII, Low-PLR and surgical intervention were 0.742 (95% CI: 0.630 ∼ 0.854, *P* = 0.003), 0.662 (95% CI: 0.521 ∼ 0.803, *P* = 0.048) and 0.730 (95% CI: 0.595∼0.866, *P* = 0.005), respectively (Figure 6). Compared with Low-PLR and surgical intervention, Low-SII showed higher value in aiding the identification of NEC-related deaths, with a sensitivity of 74.1%, a specificity of 91.7%, a PPV of 61.5%, and an NPV of 93.8% (Table 6). Referring to SII results, we can identify patients with NEC at high risk of poor clinical outcome early.

**Table 6:** Predictive value of Low-SII, Low-PLR and surgical intervention for mortality.

Variables	Sensitivity (%)	Specifificity (%)	PPV (%)	NPV (%)	+LR	−LR
Low-SII	92.86	55.56	20.00	98.48	2.09	0.13
Low-PLR	78.57	53.85	16.92	95.45	1.70	0.40
Surgical intervention	78.57	67.52	22.45	96.34	2.42	0.32

SII, systematic immuno-inflammatory index; PLR, platelet-to-lymphocyte ratio; PPV, positive predictive value; NPV, negative predictive value; +LR, positive likelihood ratio; −LR, negative likelihood ratio.

## Discussion

NEC is a common disease in neonates, especially the preterm, which begins abruptly, progresses rapidly, and has high mortality. As we know, surgical NEC has a significantly higher mortality compared to the medical treatment. Given the high mortality of surgical NEC, it is important to identify its early predictors to improve identification of high-risk patients and hence initiate timely treatment. Furthermore, most hospitals are equipped with neonatology department, but neonatal surgery department is relatively rare, especially in developing countries or regions. Early identification of patients at high risk of surgery may improve outcomes by optimizing earlier transfer to surgical centers and earlier surgery, and facilitate the rational allocation of limited medical resources. Therefore, it is necessary to further explore the early predictors for surgical NEC and develop better treatment plans for these patients.

It has recently been shown that the survival rate of premature infants (predominant incidence groups of NEC) has been rising with the development of neonatal intensive care and improvements in obstetrics, which increased the number of neonates with NEC. Studies showed that about 30% of neonates with NEC required surgical intervention, and the main surgical methods include peritoneal drainage and laparotomy (bowel resection with enterostomy formation or primary anastomosis) (3, 29). But the choice for optimal surgical method is still controversial, most surgeons generally accepted that the type of surgery is mainly based on the clinical condition of the patient as well the extent of bowel affected. As the highest mortality among neonates requiring surgery, the overall mortality of neonates with NEC is 15% and up to 34.5% in patients who received surgery, higher than that of patients treated conservatively (30). Our previous study has reported that some patients did not receive surgical intervention during the acute process of NEC, but needed surgery due to the later complications (eg, persistent intestinal obstruction or stricture), which actually had a causal relationship (18). Therefore, we included the patients who received medical treatment for NEC but received surgery for later complications of NEC during 3-month follow-up period. In our study, we found that 37.1% (49 cases) received surgical intervention, of whom 4 received peritoneal drainage and 45 received laparotomy. The overall mortality was 12.3% and 22.4% in patients who received surgery. The relatively high morbidity and low mortality may be attributed to the fact that our hospital is the largest tertiary referral center for children in southwest China, the neonates in this cohort were in relatively seriously condition and the medical technology and care relatively advanced.

Recently, there are various methods used to predict surgical NEC, among which inflammatory markers were more common because inflammatory mediators play a critical role in the occurrence of NEC. Early predictors that have been reported are as follows: C-reactive protein/albumin ratio, serum albumin concentration, interleukin (IL) 6 and coagulation function at NEC diagnosis, trends in C-reactive protein and lactate within 72 h of NEC diagnosis, IL-8 and IL-10 within 72 h of life (infants born less than 1500 g), and hyponatremia and/or the sudden decrease in plasma sodium at the onset of NEC (2, 16, 31–34). However, these methods have at least one of the following deficiencies, which limit clinical use and promotion: lack of enough sensitivity and specificity, expensive, inconvenience. As we previously reported, coagulopathy at NEC diagnosis can be used as a predictor of surgical NEC, but coagulation assessments has not been part of the routine NEC surveillance protocol at most department (16). So, the ideal early predictor should be simple, convenient, easily obtained, cheap, and non-invasive, which SII just meets.

In the study, we established an immune-inflammation-based prognostic index (SII) based on NE, LY, and PLT demonstrated that Low-SII was correlated with surgical NEC and poor clinical outcome. Regarding prediction, Low-SII at NEC diagnosis exhibited a high degree of discrimination (AUC: 0.805) for surgical NEC, outperformed Low-PLR (AUC: 0.723), with a desirable sensitivity of 87.76% and a high NPV of 90.91%. It suggested that patients with Low-SII were considered to be more likely to received surgery. It is well known that patients with NEC who receive surgery have a worse outcome than those who are treated conservatively (5, 17), as also shown in this study. Furthermore, Low-SII could identify patients at high risk of NEC-related deaths, with a high specificity of 91.7% and a NPV of 93.8%, better than variables of Low-PLR and surgical intervention. So, these findings support our hypothesis that Low-SII could be an early predictor for surgical NEC and poor clinical outcome.

SII, known as immune cells involved in inflammatory responses, is a inflammatory markers based on NE, LY, and PLT. It has been considered as a better index to reflect the local immune response and systemic inflammation. SII was first described as predictor for the prognosis of hepatocellular carcinoma (9), but it has been recently applied in inflammation-linked diseases such as acute pancreatitis and COVID-19 (12, 13). During systemic inflammation, thrombocytosis and neutrophilia are considered responses to systemic inflammation, and lymphopenia predicts cellular immune injury. Under inflammatory conditions, platelets are activated and producing large amounts of cytokines and chemokines, which promotes promoting activation of neutrophils (12). Neutrophils are the first inflammatory cells recruited to the inflammatory area, releasing proteolytic enzymes and producing reactive oxygen species for phagocytosis or bactericidal effects. At the same time, the released inflammatory mediators will damage vascular endothelial cells, which forms a vicious circle with platelet activation. Excessive stimulation of neutrophils can lead to the inflammatory cascade, ultimately triggering systemic inflammatory response syndrome. Furthermore, the abnormal stimulation will inhibit the activity of lymphocytes, resulting in decreased immune function (35). Therefore, almost all studies revealed that the increased SII value means more severe inflammatory response and higher risk of poor clinical outcome.

Interestingly, our results showed that Low-SII were associated with the higher possibility of receiving surgery and getting poor clinical outcome, different from the previous studies findings. This difference may be attributed to the different selection of study population, as this study included neonates, the majority of whom were born prematurely. The pathogenesis of NEC is complex and not well-understood, but immature intestinal host defenses are thought to play a major role in its pathogenesis. These immature defenses, included intestinal barrier function, intestinal regulation of microbial colonization, regulation of intestinal circulation, and intestinal innate and adaptive immunity, can be reflected by hematological abnormalities (14, 15).

Usually, routine inflammatory indexes such as CRP, PCT and WBC can comprehensively judge the severity of NEC. However, it is worth noting that CRP rises relatively late in the inflammatory response (between 12 and 24 h) peaking at 48 h. Serial measurements of CRP can be more help guide the clinician judge the severity of NEC and response to treatment (36). PCT was not a routine test (like general blood measurements) for neonates at high risk for NEC. Not all patients received PCT assessment at NEC diagnosis, and we did not included this marker. In our study, the difference in WBC at NEC diagnosis between surgical intervention and medical treatment groups was not statistically significant (*P* = 0.164). These indexes at NEC diagnosis were of limited value in predicting whether to receive subsequent surgical treatment. Therefore, the analysis results showed that CRP and WBC were not independent risk factors for surgical NEC, while SII index was and had considerable predictive value.

The SII value is determined by the comprehensive changes of NE, LY and PLT. In this study, patients receiving surgical intervention had lower NE (mean: 5.44 vs. 5.49, *P* = 0.921) and PLT (median: 171.0 vs. 328.0, *P* < 0.001), and higher LY (mean: 7.48 vs. 5.89, *P* = 0.016) than patients treated conservatively, which resulted in the lower calculated SII value in patient who received surgery. Neutrophils are crucial in removing pathogens, and the increased NE comprise an appropriate inflammatory response in patients with mild-moderately severe disease. However, in neonates, especially the preterm, NE is usually decreased in response to severe inflammatory stimulation due to the immaturity of immune mechanisms (37). The pathogenesis of NEC is accompanied by the disruption of intestinal mucosal barrier, resulting in the entry of toxins secreted by intestinal bacteria (the dominant Gram-negative bacterial phylum) into the bloodstream and inhibits neutrophils production from the bone marrow. Furthermore, The circulating neutrophil pool is depleted by migration to the intestine and peritoneum, and increased microvascular margins may be responsible for neutropenia (38). Neutropenia has been reported to be associated with severe NEC and poor clinical outcome (39). Lymphocytes, which play a key role in adaptive immunity, are derived from bone marrow haematopoietic stem cells and can be broadly classified as T-cells, B-cells and NK-cells. Although the role of lymphocytes in NEC remains unclear, but an overall paucity of T-cells and B-cells has been reported in surgically resected bowel affected by NEC and in murine models of NEC-like injury (38). Mu et al. reported that LY (tested within 1 week before NEC diagnosis) decreased due to the migration of activated lymphocytes to inflamed tissues and increased apoptosis of lymphocytes (38). However, no studies have reported the correlations of LY tested at NEC diagnosis with subsequent surgery and clinical outcomes. Platelets are generally considered cellular mediators of thrombosis, but also essential components of the immune system and play a direct role in regulating and inducing tissue damage and pathogen responses. Previous studies showed that the severity of thrombocytopenia correlates with the severity of inflammation in NEC, and can be a sensitive (although not specific) predictor of clinical outcomes or the need for surgical intervention (38). This study not only described the differences of NE, LY, and PLT at NEC diagnosis between the surgical and conservative groups, but also combined the three to calculate SII value for better application in clinical practice. To our knowledge, we are the first to indicate that SII has two uses, including differentiating surgical NEC and predicting clinical outcomes.

## Limitation

However, there are some limitations to this study. Firstly, inherent biases were inevitable given the retrospective nature and relatively small cohort of this single-center study. In addition, selection bias and confounding bias were inevitable since we included the neonates with relatively seriously condition in this cohort. Third, SII was calculated based on the blood routine examinations acquired within 12 h after diagnosis of NEC, which cannot comprehensively consider the influence of gestational age or postmenstrual age at NEC diagnosis on the normal range of NE, PLT, and LY. Therefore, it is still necessary and valuable to conduct a prospective multi-center study with a larger cohort to validate the preliminary results of these findings. In the future, as the research deepens, the monitoring of SII in neonates at high risk of NEC can provide effective references for formulating individualized treatment plans and evaluating clinical outcomes.

## Conclusion

To the best of our knowledge, this is the first study investigating the clinical application of SII in the neonatal population and yielded interesting results. Although retrospective design was adopted, to provide a degree of theoretical basis for the later prospective study. Our observations showed that Low-SII (SII ≤ 235.85) at NEC diagnosis has reasonably good predictive ability for surgical intervention in neonates with NEC, outperformed Low-PLR (PLR ≤ 50.17). This finding may allows the surgeon or neonatologist to optimize referral and treatment strategies, facilitate the rational allocation of limited medical resources. The study also demonstrated the predicative ability of Low-SII for mortality, outperformed Low-PLR and surgical intervention, and provided reference for parental counselling when discussing the clinical outcomes in NEC cases. In brief, SII is very attractive as monitoring tool in neonates at high risk of NEC because it is easily accessible and can be processed in a standardized fashion requiring only small blood volumes.

## Data Availability

The original contributions presented in the study are included in the article/Supplementary Material, further inquiries can be directed to the corresponding author/s.
